# The construction of recombinant *Lactobacillus casei* vaccine of PEDV and its immune responses in mice

**DOI:** 10.1186/s12917-021-02885-y

**Published:** 2021-05-04

**Authors:** Xiaowen Li, Bingzhou Zhang, Dasheng Zhang, Sidang Liu, Jing Ren

**Affiliations:** 1 Swine Research Institute of New Hope Group, Tai’an, China; 2 Shandong Swine Herd Health Big Data and Intelligent Monitoring Engineering Laboratory, Tai’an, China; 3 College of Animal Science and Technology, Shandong Agricultural University, Tai’an, China; 4 Chang Sha Axybio Bio-tech Co., Ltd, Changsha, China; 5 Shandong Provincial Key Laboratory of Biophysics, Institute of Biophysics, Dezhou University, Dezhou, 253023 China

**Keywords:** PEDV, *Lactobacillus casei* vaccine, PEDV S protein, OMP16, Immune responses

## Abstract

**Background:**

Porcine epidemic diarrhea (PED) is a contagious intestinal disease caused by porcine epidemic diarrhea virus (PEDV) characterized by vomiting, diarrhea, anorexia, and dehydration, which have caused huge economic losses around the world. At present, vaccine immunity is still the most effective method to control the spread of PED. In this study, we have constructed a novel recombinant L. casei-OMP16-PEDVS strain expressing PEDVS protein of PEDV and OMP16 protein of *Brucella abortus* strain. To know the immunogenicity of the recombinant L. casei-OMP16-PEDVS candidate vaccine, it was compared with BL21-OMP16-PEDVS-F, BL21-OMP16-PEDVS, and BL21-PEDVS recombinant protein.

**Results:**

The results showed that we could detect higher levels of IgG, neutralizing antibody, IL-4, IL-10, and INF-γ in serum and IgA in feces of L. casei-OMP16-PEDVS immunized mice, which indicated that L. casei-OMP16-PEDVS candidate vaccine could induce higher levels of humoral immunity, cellular immunity, and mucosal immunity.

**Conclusion:**

Therefore, L. casei-OMP16-PEDVS is a promising candidate vaccine for prophylaxis of PEDV infection.

**Supplementary Information:**

The online version contains supplementary material available at 10.1186/s12917-021-02885-y.

## Introduction

Porcine epidemic diarrhea (PED) is caused by porcine epidemic diarrhea virus (PEDV) with symptoms including diarrhea, vomiting, anorexia, dehydration, and weight loss in piglets [1, 2]. Pigs of all ages can be infected with different symptoms and the mortality rate in piglets is up to 100% [3], which have led to huge economic losses all around the world. To control the spread of PEDV, most kinds of vaccines are constructed, such as aluminum-hydroxide-adjuvanted inactivated vaccine, bivalent inactivated Transmissible Gastroenteritis Virus (TGEV) and PEDV vaccine, and attenuated PEDV vaccine [4]. Although these vaccines play an important role in controlling PED, they all have their defects. Inactivated vaccines cannot activate cellular immune responses, attenuated vaccine is not very safe, and they all cannot induce sufficient production of virus-specific IgA antibodies of mucosal immune responses. Therefore, it is necessary and urgent to develop a new vaccine to control PED.

*Lactobacillus casei* is often considered to be a kind of safe vector system for targeted delivery of antigens in oral immunization, with beneficial effects on the health of humans and animals [5]. Meanwhile, it can be used as a delivery system to regulate the T-helper cell response and stimulate the secretion of specific IgAs for mucosal immunity [6]. On the other hand, *Lactobacillus casei* recombinant vaccine is easier administration, lesser chance of hypersensitivity reaction, and more cost-effective compared with traditional vaccines. Based on the reports, *Lactobacillus casei* recombinant vaccines have been successfully used in the prevention and control of human papillomavirus, *Streptococcus pneumonia*, and *Escherichia coli* [7–9]. There are also some similar attempts in designing of PED vaccines. The researches find that a recombinant *Lactococcus lactis* strain expressing a variant porcine epidemic diarrhea virus S1 gene could induce high levels of IL-4 and IFN-γ in immunized mice [10]. *Lactobacillus casei*-based anti-PEDV vaccine expressing microfold cell-targeting peptide Co1 fused with the COE antigen of PEDV could also induce effective immune response [11]. To improve the effectiveness of PEDV vaccine. In this study, we construct a new *Lactobacillus casei* recombinant vaccine of PED, which can stimulate stronger mucosal, humoural and cellular immune responses against PEDV infection via oral administration.

PEDV, a member of the coronaviridae family, consisted by four structural proteins which contain the 150–220 kDa glycosylated spike (S) protein, the 20–30 kDa membrane (M) protein, the 7 kDa envelope (E) protein, and 58 kDa nucleocapsid (N) protein [12]. Thereinto, the S protein can be divided into S1 (1–735 amino acid) and S2 (736-last amino acid) domains [13], and S1 protein includes the receptor-binding region and the main neutralizing epitopes [14]. Vaccine adjuvant acts as an immunomodulator can induce and enhance immune responses against co-delivered antigens. OMP16 protein of *Brucella abortus* strain was verified that could activate dendritic cells in vivo, induces a th1 immune response, and was a promising self-adjuvanting vaccine [15–17]. Therefore, OMP16 protein was inserted into the *Lactobacillus casei* recombinant vaccine in our study.

So far, few studies about the *Lactobacillus casei* recombinant vaccine of PEDV are reported. Therefore, this study is aimed to construct a novel *Lactobacillus casei* candidate oral vaccine, which can supply better humoral immunity, cellular immunity, and mucosal immunity to prevent the spread of PED.

## Materials and methods

### Bacterial strains, viruses, culture conditions, plasmids, and primers

The bacterial strains, plasmids, and primers used in this study are listed in Table 1. The standard reference strain of *Lactobacillus casei* ATCC 393 was cultured in de Man Rogosa and Sharpe (MRS) broth at 37 °C [18]. The BL21 (DE3) and DH5α were cultured in Luria-Bertani (LB) medium at 37 °C [19]. The recombinant *Lactobacillus casei*, BL21 (DE3), and DH5α strains were cultured in the corresponding medium with proper antibiotics, respectively. *Brucella abortus* was grown in Tryptic Soy Broth (TSB) or Tryptic Soy Agar (TSA) medium (Difco Laboratories, Detroit, MI, USA) at 37 °C. The Vero cells infected with PEDV strains were cultured in DMEM (Gibco, Langley, VA, USA) supplemented with 10 μg/mL trypsin (Gibco, Langley, VA, USA) [20]. The pVE5523 and pET28a (+) plasmids were expression vectors of *Lactobacillus casei* and *Escherichia coli*, respectively.

**Table 1:** Characteristics of bacterial strains, plasmids, and primers used in this study

Strain/plasmid/primer	Characteristics and/or sequences	Source/reference
Strain		
DH5α	Genotype: supE44 ΔlacU169 (φ80lacZ△M15) hsdR17 recA1 endA1 gyrA96 thi-1 relA1	TaKaRa (Otsu, Japan)
BL21	Genotype: F-ompT hsdS (rB-mB) gal dcm (DE3)	TaKaRa (Otsu, Japan)
*Lactobacillus casei*	ATCC393, used as a vector system for targeted delivery of antigens in oral immunization.	Preserved in our lab
*Brucella abortus*	*Brucella abortus* S19, supply gene sequence of *omp16* gene	Preserved in our lab
PEDV	AJ1102, variant strain, isolated in China.	Preserved in our lab
Plasmids		
pET28a (+)	The expression vector of *Escherichia coli*, Kan^r^	Preserved in our lab
pVE5523	The expression vector of *Lactobacillus casei*, Amp^r^	BioVector NTCC
pET28a-PEDVS	The recombinant vector of pET28a and S protein of PEDV, Kan^r^	This study
pET28a-OMP16-PEDVS	The recombinant vector of pET28a and fusion protein (S protein of PEDV and OMP16 protein of *Brucella abortus*), Kan^r^	This study
pVE5523-OMP16-PEDVS	The recombinant vector of pVE5523 and fusion protein (S protein of PEDV and OMP16 protein of *Brucella abortus*), Amp^r^	This study
Primers		
PEDVS-F1/R1	F1: CCGGAATTCATGCTGAGTCATGAACAGCC R1: TGCTCTAGATTAATATGCAGCCTGCTCTG	This study
PEDVS-F2/R2	F2:GGTGGTGGCGGTAGCGGCGGTGGTGGCTCTGGTGGCGGCGGTTCTTTCTTTTGTTACTTTGCCAT R2: CGCGATATCTTAATATGCAGCCTGCTCTG	This study
OMP16-F/R	F: CGCGTCGACATGGCGTCAAAGAAGAACCTTCCG R:AGAACCGCCGCCACCAGAGCCACCACCGCCGCTACCGCCACCACCGGTACCCCGTCCGGCCCCGT	This study

### The construction of recombinant *Lactobacillus casei* and BL21 strains

The recombinant expression plasmids were constructed based on the plasmids and primers in Table 1. At first, the partial sequence of PEDV S gene (493–708 amino acid), the partial sequence of PEDV S’ gene (493–708 amino acid), and the partial sequence of *Brucella abortus* OMP16 gene (26–168 amino acid) were amplified using primer pairs PEDVS-F1/R1, PEDVS-F2/R2, and OMP16-F/R, respectively. Subsequently, the overlap extension method was used in OMP16 and PEDVS’ fragments to construct a new fragment OMP16-PEDVS with linker polypeptide (GGGGSGGGGSGGGGS) stuck in the middle. Then, the fragments PEDVS was inserted into pET28a plasmid with EcoRI/XbaI restriction enzymes to generate recombinant plasmid pET28a-PEDVS, and the new fragments OMP16-PEDVS was inserted into pET28a and pVE5523 plasmids with EcoRI/XbaI and SalI/EcoRV restriction enzymes to generate recombinant plasmid pET28a-OMP16-PEDVS and pVE5523-OMP16-PEDVS, respectively. The recombinant plasmids (pET28a-PEDVS, pET28a-OMP16-PEDVS, and pVE5523-OMP16-PEDVS) were transformed into BL21 (DE3) or *Lactobacillus casei* ATCC393 by transformation or electroporation based on the reported paper [21].

### Analysis of protein expression by western blot

The protein expression of BL21-pET28a-PEDVS, BL21-pET28a-OMP16-PEDVS, and L. casei-pVE5523-OMP16-PEDVS strains were detected based on the reported method with some modification [21–23]. The recombinant strains BL21-pET28a-PEDVS and BL21-pET28a-OMP16-PEDVS were cultured in LB broth and IPTG was used to harvest to pET28a-PEDVS and pET28a-OMP16-PEDVS proteins. The blank vector was used as a negative control. After cell lysis and centrifugation, the supernatant and sediment were collected. Then, the target proteins were purified using the Nickel affinity chromatography column based on the previous study [24]. Meanwhile, the cultural supernatant of the recombinant L. casei-pVE5523-OMP16-PEDVS strain was also harvested by centrifugation at 9000×g for 10 min at 4 °C. Whereafter, the samples with sodium dodecyl sulfate (SDS) loading buffer were boiled 10 mins. The proteins were separated by 12% sodium dodecyl sulfate-polyacrylamide gel electrophoresis (SDS-PAGE) and then transferred into PVDF membranes (Millipore, Mississauga, ON, Canada). Membranes were blocked with 5% skimmed milk for 2 h at 37 °C and then incubated with murine monoclonal antibody of S protein overnight at 4 °C and HRP conjugated goat anti-mouse IgG (ABclonal, Wuhan, China) for 2 h at 37 °C. The protein bands were visualized using the Clarity™ Western ECL Blotting Substrate (Bio-Rad, Hercules, CA, USA).

### Immunization and sample collection

The immunogenicity of recombinant *Lactobacillus casei* vaccine was evaluated using six-week-old female specific pathogen-free (SPF) BALB/c mice [10, 18], which were purchased from Shandong Agricultural University animal center. A total of 50 mice were randomly divided into 5 groups with 10 mice in each group. The mice were immuned with recombinant *Lactobacillus casei* vaccine and purified protein (pET28a-PEDVS, pET28a-OMP16-PEDVS, pET28a-OMP16-PEDVS+Freund’s complete adjuvant), respectively. The immunization protocol was performed based on Table 2 and a booster immunization was given after 13 days.

**Table 2:** The immune protocol of BALB/c mice

Grouping	Characteristics	Immune methods	Immunizing dose
L. casei-OMP16-PEDVS	Recombinant strain *L. casei* contains pVE5523-OMP16-PEDVS vector	PO	200 μL
BL21-PEDVS	pET28a-PEDVS protein	IM	200 μg
BL21-OMP16-PEDVS	pET28a-OMP16-PEDVS protein	IM	200 μg
BL21-OMP16-PEDVS-F	pET28a-OMP16-PEDVS protein with Freund’s complete adjuvant	IM	200 μg
PBS	Negative control	IM	200 μL

For the serological study, serum was collected on 0, 14, and 28 days post-immunization (dpi) via tail vein punching and stored at − 20 °C until use. Feces were collected 1 day before vaccination and every boosting time. For IgA detection, feces were diluted (w/v) with 0.05 M sodium EDTA at 1:4 ratios just after collection and incubated for 14 h at 4 °C following proper mixing. The supernatant was collected by 12,000×g centrifugation and preserved at − 20 °C until use. At 28th dpi; three mice from each group were sacrificed and the intestine was processed for IgA detection according to the author described previously [10, 18, 23].

### Determination analysis of antibody levels

The levels of IgG in the sera and IgA in the feces were measured by the ELISA methods with some modification [18, 23]. The methods were as follows: Polystyrene microliter plates were coated overnight at 4 °C with 100 μL 10 μg/mL PEDVS protein, OMP16-PEDVS protein, or 100 μL recombinant L. casei-OMP16-PEDVS strain. After blocking with 5% skimmed milk, the collected samples were serially diluted in PBS, added in triplicate, and incubated at 37 °C for 1 h. Then, an HRP conjugated goat anti-mouse IgG or IgA antibody (Invitrogen, USA) was added to each well (1:5000) and incubated for 1 h at 37 °C. The polystyrene microtiter plates were washed 5 times during each step. At last, 100 μL of TMB substrate (tetramethylbenzidine and H_2_O_2_) was added to each well and 50 μL of stop solution was added after 10 mins. The OD values at 630 nm were measured using a multimode plate reader (EnVision).

### Virus neutralization assays

The neutralizing antibody titers of PEDV in sera were examined according to the methods with some modifications [25, 26]. Briefly, the murine serum was heat-inactivated (56 °C for 30 min) and then serially two-fold diluted in 96 well plates (Corning, USA) with triplicates of each sample. Then, an equal volume of 200 TCID_50_ / 50 μL PEDV strains were added to 96 well plates and incubated for 1 h at 37 °C. The mixture was added to new 96 well plates coated with Vero cell monolayers and incubated for 1 h at 37 °C. Cells were then washed and incubated in the maintenance medium at 37 °C in 5% CO_2_. After 2 days, the cytopathic effect (CPE) was observed using an inverted microscope and the neutralizing concentration was defined as the lowest concentration of antibodies in the serum.

### Cytokine detection

To detect the secretion of cytokines, supernatants were obtained from the laboratory mice (0, 14, and 28 days). Levels of secreted IL-4, IL-10, and IFN-γ were determined using commercial ELISA kits (Elabscience Biotechnology Co., Ltd., Wuhan) according to the manufacturer’s recommendations, respectively. Cytokine was quantified from the different standard curves prepared from standard reagents provided by the kits respectively and optical density (OD) value was detected at 450 nm from each plate using a multimode plate reader (EnVision) [23, 27].

### Statistical analysis

All data were obtained from at least three independent experiments, and results were presented as the means ± standard deviation (SD). The statistical analysis was performed using two-tailed *t*-tests and one-way analysis in Graph Pad Prism 7.0 (GraphPad Software Inc., USA). The significant difference was defined as ∗ *p* < 0.05, and the various degrees of significant difference were designated as ∗∗ *p* < 0.01, ∗∗∗ *p* < 0.001, respectively.

## Results

### The verification of recombinant *Lactobacillus casei* and BL21 strains

**Fig. 1:**
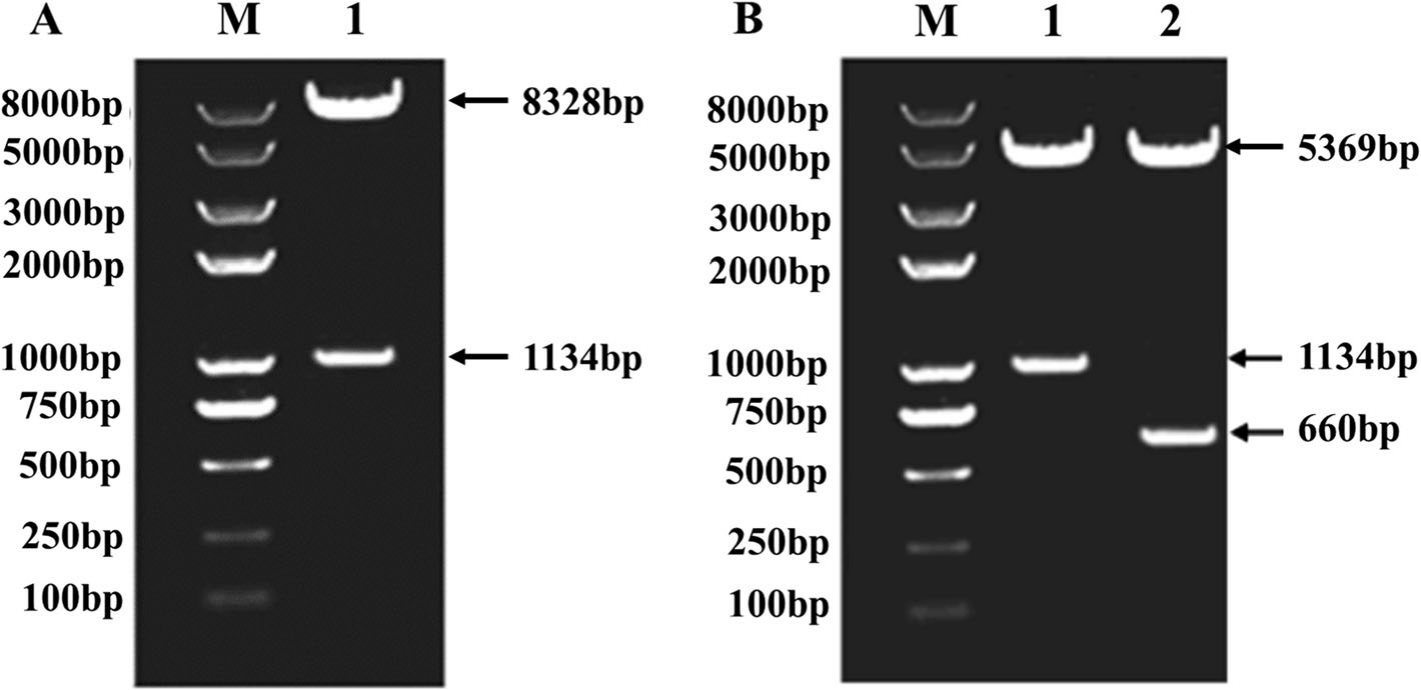
The enzyme digestion results of recombinant plasmids. A: The enzyme digestion results of the pVE5523-OMP16-PEDVS plasmid. M: D8000 DNA Ladder Marker; 1: pVE5523-OMP16-PEDVS plasmid. B: The enzyme digestion results of pet28a-OMP16-PEDVS and pET28a-PEDVS plasmid. M: D8000 DNA Ladder Marker; 1: pet28a-OMP16-PEDVS plasmid; 2: pET28a-PEDVS plasmid

To verify the constructed recombinant plasmids, the recombinant expression plasmids pET28a-PEDVS, pET28a-OMP16-PEDVS, and pVE5523-OMP16-PEDVS were digested using NcoI/XhoI restriction enzymes, and the enzyme digestion results were shown in Fig. 1. The sizes of target bands in electrophoretograms were the same as the expected results and sequencing results indicated that the recombinant expression plasmids exhibited no mutation. These results indicated the successful construction of pET28a-PEDVS, pET28a-OMP16-PEDVS, and pVE5523-OMP16-PEDVS recombinant plasmids.

**Fig. 2:**
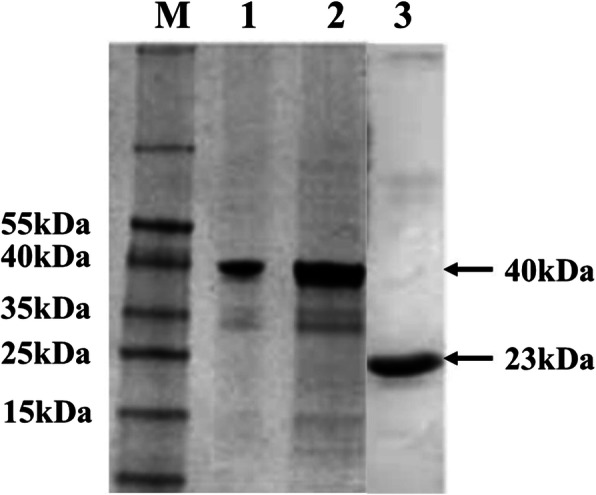
The verification of recombinant expression proteins. M: protein marker; 1: the secretory protein form recombinant L. casei-pVE5523-OMP16-PEDVS strain; 2: the purified protein from BL21-pET28a-OMP16-PEDVS strain; 3: the purified protein from BL21-pET28a-PEDVS strain

The results of western blot showed that the recombinant proteins pET28a-OMP16-PEDVS and pET28a-PEDVS were expressed in the supernatant of BL21-pET28a-OMP16-PEDVS and BL21-pET28a-PEDVS strains, respectively. The pVE5523-OMP16-PEDVS protein was also verified in the cultural supernatant of L. casei-pVE5523-OMP16-PEDVS strain. The specific bands from Fig. 2 showed that the recombinant proteins pET28a-OMP16-PEDVS, pVE5523-OMP16-PEDVS, and pET28a-PEDVS were all harvested successfully (Fig. 2).

### The IgG antibody levels in serum of mice immunized with candidate vaccines

**Fig. 3:**
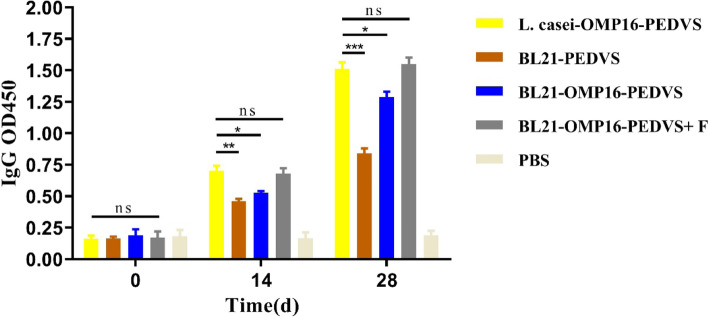
The IgG antibody levels of candidate vaccines in the serum of immunized mice. Serum was collected on days 0, 14, and 28 days before or after immunization and examined via commercial ELISA kits and measured at an absorbance of 450 nm. Bars represent the mean ± standard deviation of three independent experiments. * *p* < 0.05, ** *p* < 0.01, and **** *p* < 0.0001 represent increasing degrees of significant differences, respectively, and ns means no significant difference

To evaluate the specific immunogenicity of generated vaccine candidates, BALB/c mice were selected and divided into 5 groups. Then, the levels of IgG in the serum and IgA in the feces were measured with commercial ELISA kits. The results revealed that there were no substantial differences for IgG levels among the vaccinated groups and almost no IgG antibody was found in all mice before immunization. However, substantial differences were subsequently found after the first vaccination and IgG antibody levels in serum of 28 days were obviously higher than that of in 14 days. Among 5 group mice, the mice immunized with L. casei-OMP16-PEDVS and BL21-OMP16-PEDVS-F showed similar and highest immunogenicity. Therefore, L. casei-OMP16-PEDVS and BL21-OMP16-PEDVS-F could produce highest immunogenicity, followed by BL21-OMP16-PEDVS and BL21-PEDVS (Fig. 3).

### The IgA antibody levels in feces of mice immunized with candidate vaccines

**Fig. 4:**
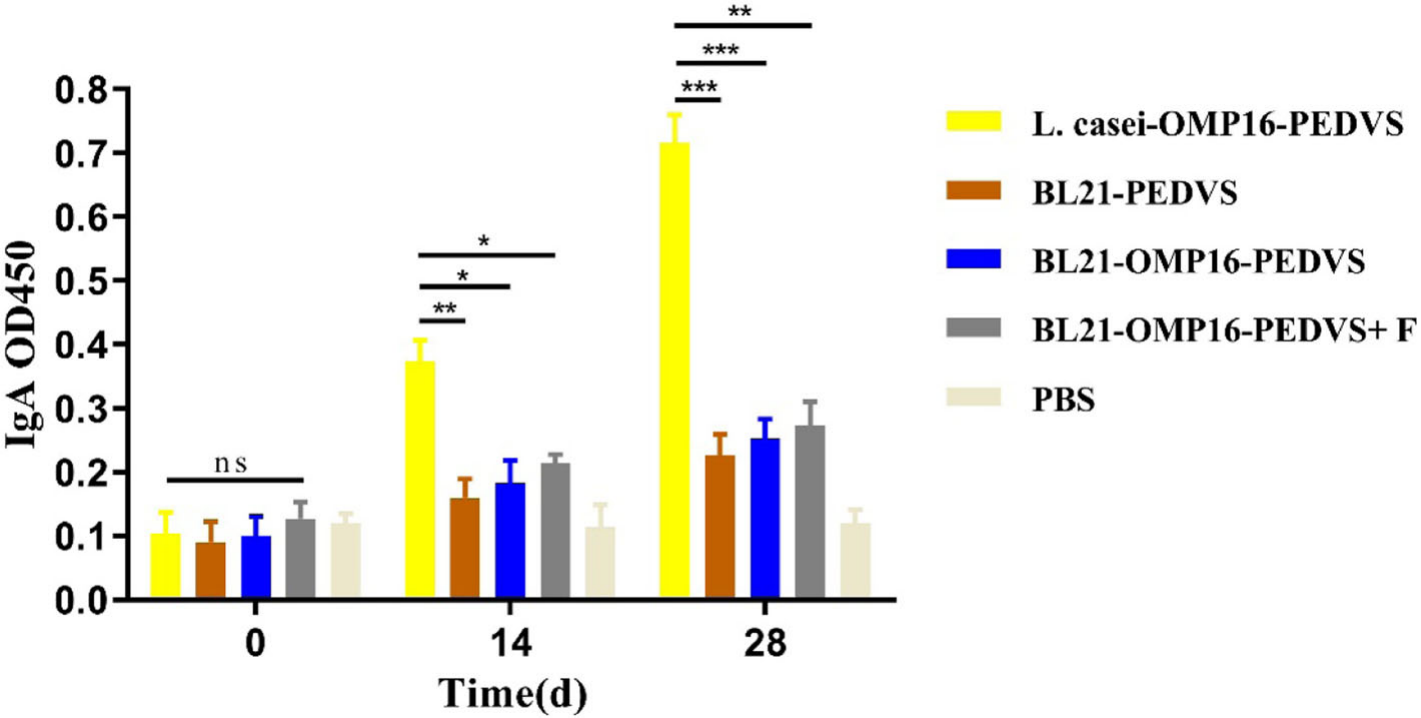
The IgA antibody levels of candidate vaccines in feces of immunized mice. Feces were collected on day 0, 14, and 28 days before or after immunization and examined via commercial ELISA kits and measured at an absorbance of 450 nm. Bars represent the mean ± standard deviation of three independent experiments. * *p* < 0.05, ** *p* < 0.01, and **** *p* < 0.0001 represent increasing degrees of significant differences, respectively, and ns means no significant difference

To evaluate the specific immunogenicity of generated vaccine candidates, the levels of IgA antibody in feces of mice were also evaluated. The results showed that there was no special anti-PEDVS IgA antibody existed before immunization. However, large amounts of IgA antibody in feces of L. casei-OMP16-PEDVS immunized mice were detected and it was obviously higher than that of in BL21-OMP16-PEDVS-F, BL21-OMP16-PEDVS, and BL21-PEDVS group mice at 14 days after immunization. At 28 days after immunization, the IgA antibody levels of L. casei-OMP16-PEDVS immunized mice reached its highest maximum. Meanwhile, the IgA antibody levels in the other three groups did not present an obvious increase. Therefore, the candidate vaccine L. casei-OMP16-PEDVS could stimulate higher levels of antibody in immunized mice compared with BL21-OMP16-PEDVS-F, BL21-OMP16-PEDVS, and BL21-PEDVS immunized mice (Fig. 4).

### The neutralizing antibody levels of serum in immunized mice

**Fig. 5:**
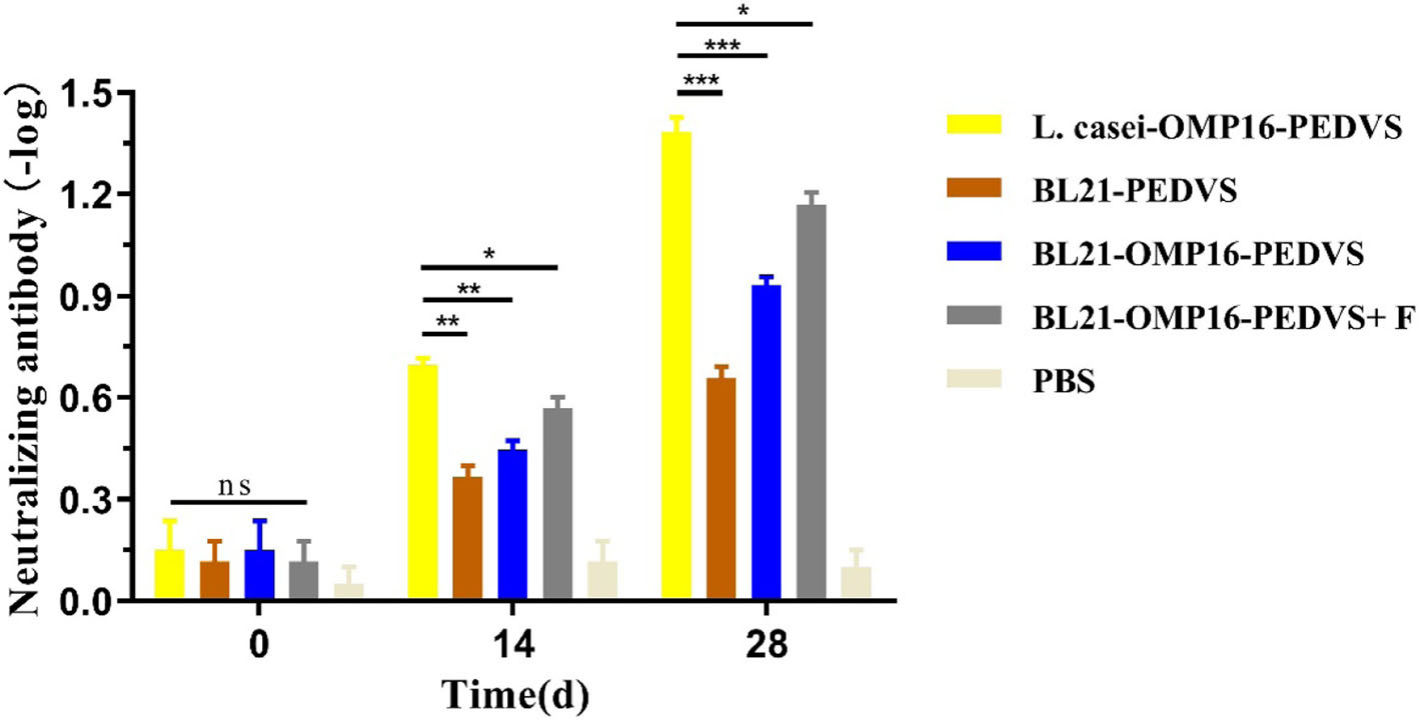
The neutralizing antibody levels of candidate vaccines in the serum of immunized mice. Serum was collected on days 0, 14, and 28 days before or after immunization and examined via neutralization test. Bars represent the mean ± standard deviation of three independent experiments. * *p* < 0.05, ** *p* < 0.01, and **** *p* < 0.0001 represent increasing degrees of significant differences, respectively, and ns means no significant difference

To evaluate the protective effect of candidate vaccines of L. casei-OMP16-PEDVS, BL21-OMP16-PEDVS-F, BL21-OMP16-PEDVS, and BL21-PEDVS in mice, the neutralizing antibody levels were measured. Results showed that no neutralizing antibody was detected before immunization. Neutralizing antibody was detected at 14 days after immunization and it increased at 14 days after booster immunization. The antibody response in mice that received L. casei-OMP16-PEDVS possessed a stronger anti-PEDV neutralizing activity than that in mice orally administered with BL21-OMP16-PEDVS-F, BL21-OMP16-PEDVS, and BL21-PEDVS. Therefore, the candidate vaccine L. casei-OMP16-PEDVS could stimulate highest neutralizing antibody level, followed by BL21-OMP16-PEDVS-F, BL21-OMP16-PEDVS, and BL21-PEDVS (Fig. 5).

### Cytokine levels

**Fig. 6:**
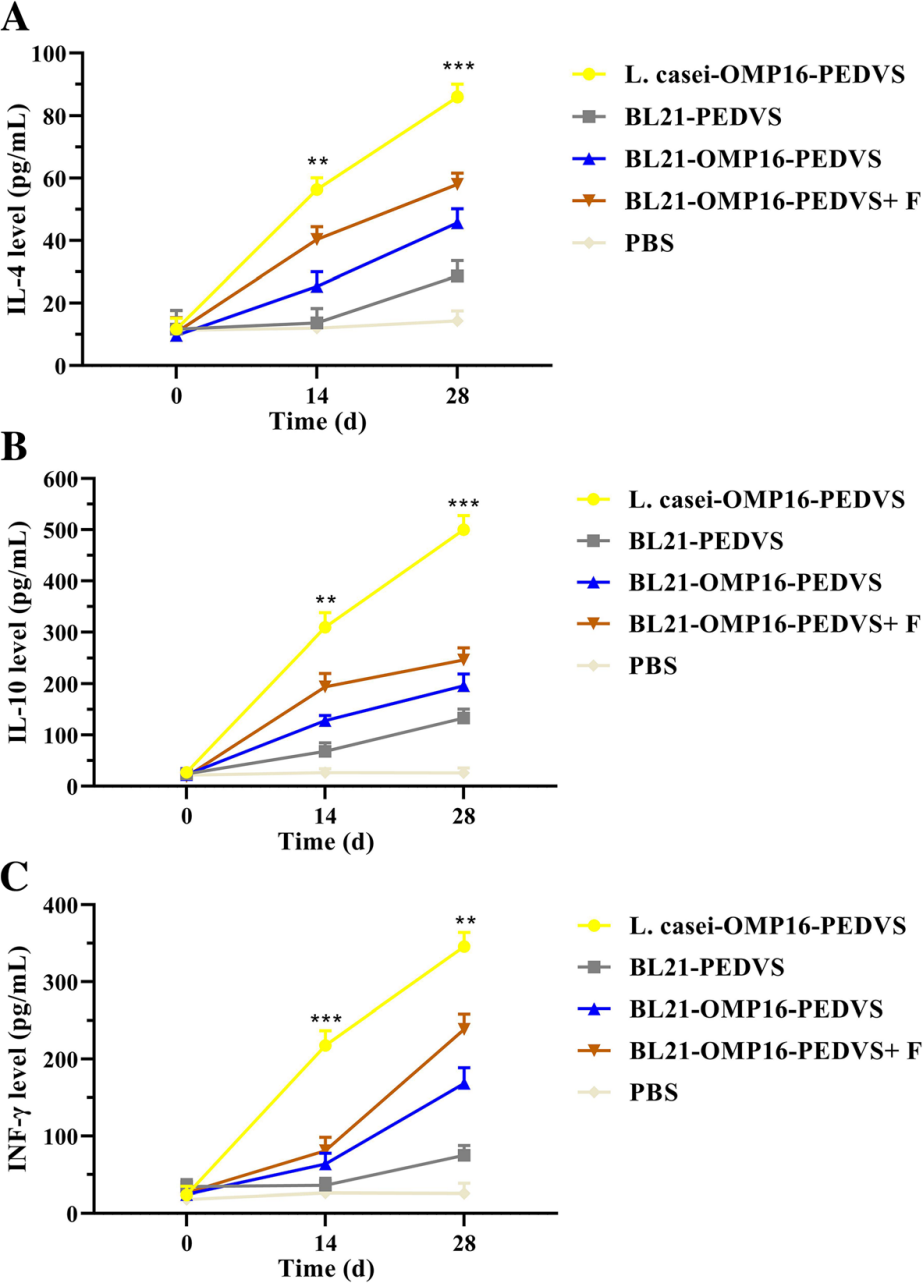
Detection of cytokine levels from the serum of immunized mice. Serum was collected on days 0, 14, and 28 days before or after immunization and examined via commercial ELISA kits. The absorbance value was measured at an absorbance of 450 nm for IL-4 (**a**), IL-10 (**b**), and IFN-γ (**c**), respectively. Bars represent the mean ± standard deviation of three independent experiments. * *p* < 0.05, ** *p* < 0.01, and **** *p* < 0.0001 represent increasing degrees of significant differences, respectively

To compare the cellular immune response level of L. casei-OMP16-PEDVS, BL21-OMP16-PEDVS-F, BL21-OMP16-PEDVS, and BL21-PEDVS immunized mice, IL-4, IL-10, and IFN-γ were determined, respectively. The results showed that the levels of cytokines IL-4, IL-10, and IFN-γ in the sera of mice were all very low and have no significant difference before immunization. Whereas, similar changes were observed in the results of IL-4, IL-10, and IFN-γ. At 14 days after immunization, the level of IL-4, IL-10, and IFN-γ in L. casei-OMP16-PEDVS immunized mice were higher than BL21-OMP16-PEDVS-F, BL21-OMP16-PEDVS, and BL21-PEDVS immunized mice. At 14 days after the booster immunization, a higher IL-4, IL-10, and IFN-γ level in L. casei-OMP16-PEDVS immunized mice were detected compared with that of in other three groups. Therefore, the candidate vaccine L. casei-OMP16-PEDVS could stimulate highest IL-4, IL-10, and IFN-γ level, followed by BL21-OMP16-PEDVS-F, BL21-OMP16-PEDVS, and BL21-PEDVS (Fig. 6).

## Discussion

Since a large-scale outbreak of PED that caused by PEDV variants occurred in October 2010, which has resulted in tremendous economic losses in China and all around the world [1]. However, traditional vaccines are all designed based on CV777 classical strain which cannot supply sufficient protection to PEDV variant [4]. To control the spread of PEDV and reduce the economic losses, novel vaccines of PEDV variant strains are also designed. At present, PEDV inactivated vaccine and attenuated vaccine of PEDV variant strains are all approved by Chinese government and there are all exhibiting promising prospects in controlling PED. But defects are also existed in the two kinds of novel vaccines. PEDV infects swine through the digestive tract and has intestinal tissue tropism. Therefore, Mucosal immunity is more effective than systemic immunity in preventing PEDV entry into intestinal epithelial cells, and vaccines must provide mucosal protection effectively in the intestinal tract. So, In this study, we construct a new kind of vaccine which can stimulate stronger anti-PEDV-specific IgG and sIgA antibodies.

*Lactobacillus casei* has potential immune-modulatory properties as a vaccine delivery vehicle and the expression of bioactive compounds on the cell wall of this bacterium can stimulate appropriate immune responses [28, 29]. It is widely used for expressing several heterologous antigens of human papillomavirus, *Streptococcus pneumonia*, and *Escherichia coli* as vaccines in animal models, which all showed excellent immunogenicity [7–9]. Compared with the inactivated vaccine and attenuated vaccine, the *Lactobacillus casei* vector vaccine can also stimulate higher IgA level and cellular immune response. Studies have shown that IgA’s first line of defense in the intestine would be better than IgG in protecting piglets from PEDV infection [10, 30]. Therefore, it is promising to develop a kind of *Lactobacillus casei* vector vaccine of PED.

Based on the reports, the S protein of PEDV can be divided into S1 (1–735 amino acid) and S2 (736-last amino acid) domains [13], and S1 protein includes the receptor-binding region and the main neutralizing epitopes [14]. The core neutralizing epitope (COE) can induce strong neutralizing antibodies against PEDV [31, 32]. Combining with the antigenicity analysis, a partial sequence of the S1 gene (1477–2124 bp) was selected to construct the recombinant plasmid. The selected small fragment was proved that not only had good immunogenicity but also contributed to the secreted expression of *Lactobacillus casei*. On the other hand, *Pasquevich* found that *Brucella abortus* outer membrane protein 16 could activate dendritic cells in vivo, induces a th1 immune response, and was a promising self-adjuvanting vaccine against systemic and oral acquired brucellosis [15]. Similar research that unlipidated outer membrane protein omp16 from Brucella spp. as nasal adjuvant could induce a th1 immune response and modulates the th2 allergic response to cow’s milk proteins was also proved [16]. Therefore, a partial sequence of the *omp16* gene was chosen to construct recombinant plasmid to enhance to immune function in our study.

To know whether the novel *Lactobacillus casei* recombinant vaccine could induce humoral immune responses, the IgG, IgA, and neutralizing antibody levels were measured. The IgG antibody level of L. casei-OMP16-PEDVS recombinant vaccine immunized mice had no significant difference with BL21-OMP16-PEDVS-F recombinant vaccine immunized mice. But the IgA and neutralizing antibody levels were obviously higher than that of in BL21-OMP16-PEDVS-F, BL21-OMP16-PEDVS, and BL21-PEDVS recombinant vaccine immunized mice. The results showed that L. casei-OMP16-PEDVS could induce stronger humoral immune responses, especially IgA antibody level. Studies have shown that IgA was the first line of defense in the intestine and would be better than IgG in protecting piglets from PEDV infection [30]. The research also verified the result that L. casei-OMP16-PEDVS recombinant vaccine could supply better immunological protection to PEDV.

To explore the type of immune response induced by recombinant L. casei-OMP16-PEDVS, the levels of IL-4, IL-10, and IFN-γ were detected to evaluate the activity of T lymphocytes. Based on the report, IFN-γ plays an important role in cellular immune response caused when pathogens invade the body, IL-4 plays an important role in the humoral immune response and promoting immune tolerance and mucosal immunity [33], IL-10 plays essential roles in fighting against mucosal microbial infection and maintaining mucosal barrier integrity within the intestine [34]. Meanwhile, results of cytokine detection showed that the mice immunized with L. casei-OMP16-PEDVS recombinant vaccine could induce stronger expression of IL-4, IL-10, and IFN-γ, which supported the results that L. casei-OMP16-PEDVS recombinant vaccine could induce stronger humoral immune response, IgA antibody level, and cellular immune response, respectively.

## Conclusion

In summary, L. casei-OMP16-PEDVS, BL21-OMP16-PEDVS-F, BL21-OMP16-PEDVS, and BL21-PEDVS candidate vaccines were constructed in this study. Meanwhile, the humoral immune response and cellular immune response levels of these candidate vaccines in mice were evaluated. The results showed that the mice immunized with L. casei-OMP16-PEDVS could produce higher levels of IgG, IgA, neutralizing antibody, IL-4, IL-10, and INF-γ compared with the mice immunized with BL21-OMP16-PEDVS-F, BL21-OMP16-PEDVS, and BL21-PEDVS. Therefore, the recombinant L. casei-OMP16-PEDVS candidate vaccine may establish the ground for the development of a safe, effective, and convenient recombinant mucosal vaccine for prophylaxis of PEDV infection.

## Supplementary Information

**Additional file 1.**
