# Supplementary material for: The construction of recombinant Lactobacillus casei vaccine of PEDV and its immune responses in mice
Source: BMC Vet Res. 2021 May 4;17:184. doi: 10.1186/s12917-021-02885-y (PMC8097780; doi:10.1186/s12917-021-02885-y)
Supplement: Supplementary file 1 — Additional file 1. [file 12917_2021_2885_MOESM1_ESM.docx]

**1. The construction of recombinant plasmids**

① Omp16+PEDVS1 Fusion nucleotide sequence:

ATGGCGTCAAAGAAGAACCTTCCGAATAATGCCGGTGATCTGGGTCTCGGTGCAGGCGCTGCAACGCCGGGCTCCTCGCAGGACTTCACCGTTAATGTCGGCGACCGCATCTTCTTCGATCTCGATTCGTCGCTGATCCGCGCCGATGCGCAGCAGACGCTTTCCAAGCAGGCCCAGTGGTTGCAGCGTTATCCGCAGTATTCGATCACGATCGAAGGCCATGCCGACGAGCGCGGCACGCGTGAGTACAACCTCGCCCTTGGCCAGCGCCGTGCTGCCGCCACCCGCGACTTCCTCGCTTCGCGCGGTGTGCCGACCAACCGCATGCGCACCATTTCCTACGGTAATGAGCGCCCGGTTGCCGTCTGCGATGCCGACACATGCTGGTCGCAGAACCGTCGCGCCGTCACCGTTCTCAACGGGGCCGGACGGGGTACCGGTGGTGGCGGTAGCGGCGGTGGTGGCTCTGGTGGCGGCGGTTCTCTGAGTCATGAACAGCCAACTTCTTTTGTTACTTTGCCATCATTTAATGATCATTCTTTTGTTAACATTACTGTCTCTGCGTCTTTTGGTGGTCATAGTGGTGCCAACCTTATTGCATCTGACACTACTATCAATGGGTTTAGTTCTTTCTGTGTTGACACTAGACAATTTACCATTTCACTGTTTTATAACGTTACAAACAGTTATGGTTATGTGTCTAAATCACAGGACAGTAATTGCCCTTTCACCTTGCAATCTGTTAATGATTACCTGTCTTTTAGCAAATTTTGTGTTTCCACCAGCCTTTTGGCTAGTGCCTGTACCATAGATCTTTTTGGTTACCCTGATTTTGGTAGTGTTGTTAAGTTTACGTCCCTTTACTTTCAATTCACAAAGGGTGAGTTGATTACTGGCACGCCTAAACCACTTGAAGGTGTCACGGACGTTTCTTTTATGACTCTGGATGTGTGTACCAAGTATACTATCTATGGCTTTAAAGGTGAGGGTATCATTACCCTTACAAATTCTAGCTTTTTGGCAGGTGTTTATTACACATCTGATTCTGGACAGTTGTTAGCTTTTAAGAATGTCACTAGTGGTGCTGTTTATTCTGTTACGCCTTGTTCTTTTTCAGAGCAGGCTGCATATTAA

② Omp16 (26-168 amino acid) nucleotide sequence:

ATGGCGTCAAAGAAGAACCTTCCGAATAATGCCGGTGATCTGGGTCTCGGTGCAGGCGCTGCAACGCCGGGCTCCTCGCAGGACTTCACCGTTAATGTCGGCGACCGCATCTTCTTCGATCTCGATTCGTCGCTGATCCGCGCCGATGCGCAGCAGACGCTTTCCAAGCAGGCCCAGTGGTTGCAGCGTTATCCGCAGTATTCGATCACGATCGAAGGCCATGCCGACGAGCGCGGCACGCGTGAGTACAACCTCGCCCTTGGCCAGCGCCGTGCTGCCGCCACCCGCGACTTCCTCGCTTCGCGCGGTGTGCCGACCAACCGCATGCGCACCATTTCCTACGGTAATGAGCGCCCGGTTGCCGTCTGCGATGCCGACACATGCTGGTCGCAGAACCGTCGCGCCGTCACCGTTCTCAACGGGGCCGGACGGGGTACC

③ Linker nucleotide sequence:

GGTGGTGGCGGTAGCGGCGGTGGTGGCTCTGGTGGCGGCGGTTCT

④ PEDVS (493-708 amino acid) nucleotide sequence:

TTCTTTTGTTACTTTGCCATCATTTAATGATCATTCTTTTGTTAACATTACTGTCTCTGCGTCTTTTGGTGGTCATAGTGGTGCCAACCTTATTGCATCTGACACTACTATCAATGGGTTTAGTTCTTTCTGTGTTGACACTAGACAATTTACCATTTCACTGTTTTATAACGTTACAAACAGTTATGGTTATGTGTCTAAATCACAGGACAGTAATTGCCCTTTCACCTTGCAATCTGTTAATGATTACCTGTCTTTTAGCAAATTTTGTGTTTCCACCAGCCTTTTGGCTAGTGCCTGTACCATAGATCTTTTTGGTTACCCTGATTTTGGTAGTGTTGTTAAGTTTACGTCCCTTTACTTTCAATTCACAAAGGGTGAGTTGATTACTGGCACGCCTAAACCACTTGAAGGTGTCACGGACGTTTCTTTTATGACTCTGGATGTGTGTACCAAGTATACTATCTATGGCTTTAAAGGTGAGGGTATCATTACCCTTACAAATTCTAGCTTTTTGGCAGGTGTTTATTACACATCTGATTCTGGACAGTTGTTAGCTTTTAAGAATGTCACTAGTGGTGCTGTTTATTCTGTTACGCCTTGTTCTTTTTCAGAGCAGGCTGCATATTAA

Note: Yellow regions are restriction Enzyme cutting site or termination codon.

⑤ Fusion protein:

MASKKNLPNNAGDLGLGAGAATPGSSQDFTVNVGDRIFFDLDSSLIRADAQQTLSKQAQWLQRYPQYSITIEGHADERGTREYNLALGQRRAAATRDFLASRGVPTNRMRTISYGNERPVAVCDADTCWSQNRRAVTVLNGAGRGTGGGGSGGGGSGGGGSLSHEQPTSFVTLPSFNDHSFVNITVSASFGGHSGANLIASDTTINGFSSFCVDTRQFTISLFYNVTNSYGYVSKSQDSNCPFTLQSVNDYLSFSKFCVSTSLLASACTIDLFGYPDFGSVVKFTSLYFQFTKGELITGTPKPLEGVTDVSFMTLDVCTKYTIYGFKGEGIITLTNSSFLAGVYYTSDSGQLLAFKNVTSGAVYSVTPCSFSEQAAY

Note: Yellow region is linker.

**2. The IgG antibody levels in serum of mice immunized with candidate vaccines**

**3. The IgA antibody levels in serum of mice immunized with candidate vaccines**

**4. The** **neutralizing antibody levels of serum in immunized mice**

**5. Cytokine Levels**
